# Lemierre's syndrome presented with acute pancreatitis

**DOI:** 10.1002/ams2.314

**Published:** 2017-10-20

**Authors:** Laura Garcia, Kaori Ito

**Affiliations:** ^1^ Department of Emergency Medicine Henry Ford Wyandotte Hospital Wyandotte MI USA; ^2^ Department of Surgery Division of Acute Care Surgery/Surgical Critical Care Henry Ford Hospital Detroit MI USA; ^3^ Department of Emergency Medicine Division of Acute Care Surgery Teikyo University School of Medicine Tokyo Japan

**Keywords:** acute pancreatitis, *Fusobacterium necrophorum*, Lemierre's syndrome

## Abstract

**Case:**

Lemierre's syndrome is a rare clinical condition that is characterized by infected internal jugular vein thrombosis with metastatic septicemia. The most common causative agent is *Fusobacterium necrophorum*. A previously healthy 37‐year‐old woman presented to our emergency department with nausea, vomiting, and diarrhea. She was admitted to the general practice unit with a diagnosis of acute pancreatitis then was subsequently transferred to the intensive care unit due to shock. Physical examination revealed tenderness on right side of the neck. Blood cultures were remarkable for *F. necrophorum*. Clinical symptoms led to subsequent ultrasound and computed tomography scan of the neck, confirming internal jugular vein thrombosis.

**Outcome:**

The patient was treated with antibiotics for 6 weeks. Anticoagulation therapy was initiated.

**Conclusion:**

We report a case of Lemierre's syndrome that presented as acute pancreatitis. The high index of suspicion of this disease is important for early diagnosis.

## Background

Lemierre's syndrome (LS) is a septic thrombophlebitis of the internal jugular vein (IJV) caused by anaerobic bacteria, most commonly a Gram‐negative, non‐spore‐forming obligate anaerobic bacterium known as *Fusobacterium necrophorum*, which is part of the normal oral flora.^1^ Typical symptoms are sore throat, lateral neck pain, pulmonary symptoms, and fever. Next, there is invasion of the lateral pharyngeal wall and thrombophlebitis of the internal jugular vein. This is then followed by bacteremia and septic emboli to vital organs, which can lead to metastatic abscesses, as well as other fatal complications.^2^, ^3^, ^4^

It has been previously called “the forgotten disease” due to its drop in reported infections, but over the past 15 years there has been an increase in reported cases.^5^ In this modern era, the incidence is approximately 0.6–3.2 persons per million per year.^2^, ^3^, ^6^ It is most common in young adults aged between 15 and 24 years.^2^ The recent systematic review reported a pooled mortality rate of 5%.^2^

Due to its rarity, the diagnosis of this disease could be missed if we don't have a high index of suspicion. We experienced a case which initially presented like acute pancreatitis and eventually found to be LS.

## Case

**Figure 1:**
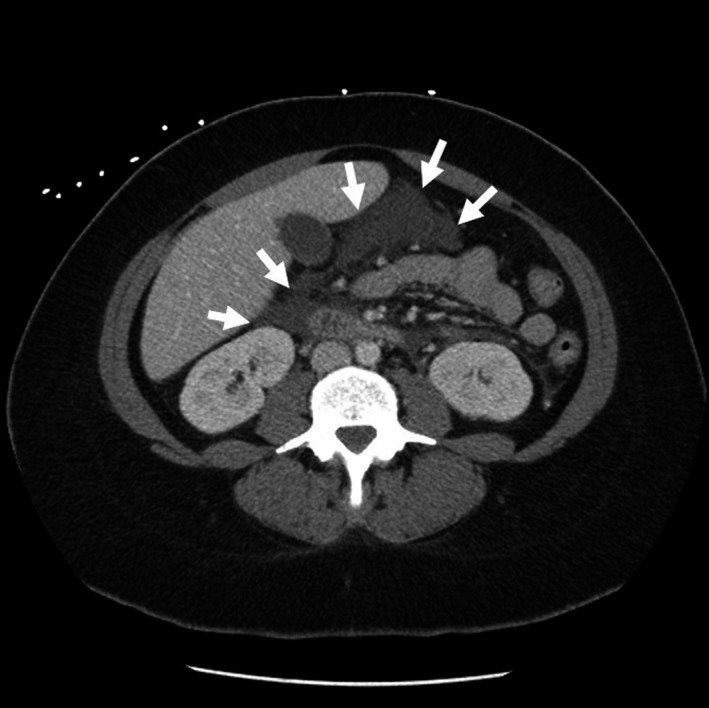
Computed tomography scan of the abdomen of a 37‐year‐old woman with nausea, vomiting, and diarrhea. The scan, on hospital day 1, revealed a peripancreatic fluid collection (arrows) compatible with acute pancreatitis.

A 37‐year‐old African American healthy immunocompetent woman presented to an emergency department with chief complaints of abdominal pain, anorexia, cough, nausea, vomiting, and diarrhea for 5 days. She admitted to tobacco use, drinking two to three alcoholic drinks two to three times per week, and denied substance abuse. Of note, the patient had not been drinking alcohol for the week prior to her presentation due to feeling unwell. Review of systems was significant for neck pain, productive cough with blood tinged sputum, fatigue, diffuse abdominal pain, nausea, vomiting, and diarrhea. Her vital signs were normal except for tachycardia (heart rate, 110 b.p.m.). On physical examination, she had diffuse tenderness to palpation on abdomen. Laboratory tests were significant for leukocytosis with bandemia (16.7 K/μL, band 15%) and elevated lipase (>2,250 IU/L). Ultrasound of the abdomen was unremarkable. Computed tomography (CT) of the abdomen and pelvis was significant for pancreatitis with a small amount of ascites (Fig. 1). Based on these findings, patient was diagnosed with acute pancreatitis. At this point, the etiology of pancreatitis was unclear because the patient had not had alcohol for 1 week. She was transferred for admission to a general medical unit for the treatment of acute pancreatitis with unknown etiology.

**Figure 2:**
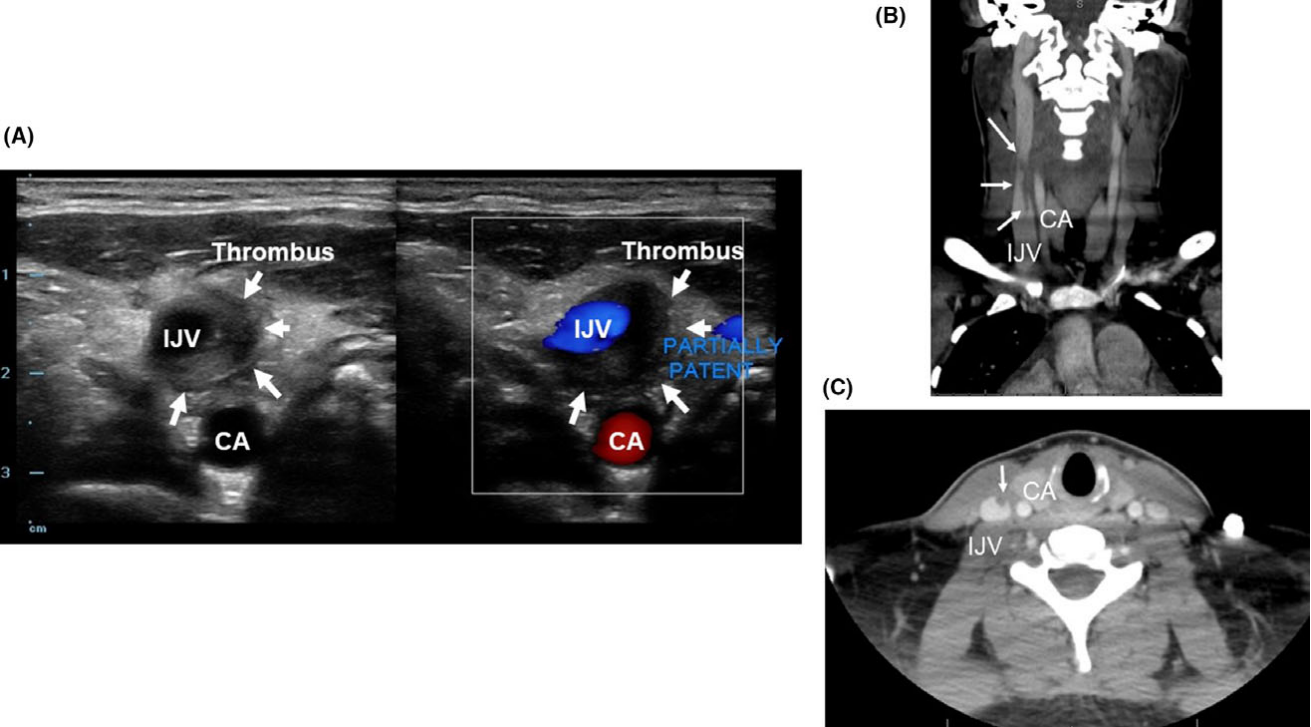
Venous duplex ultrasound (A) of a 37‐year‐old woman revealed acute thrombus (arrows) in right internal jugular vein (IJV) with partial patency. The coronal view (B) and axial view (C) on computed tomography scans of the neck revealed a thrombus within the right IJV at the level of the thyroid extending cranially (arrows). CA, carotid artery.

**Figure 3:**
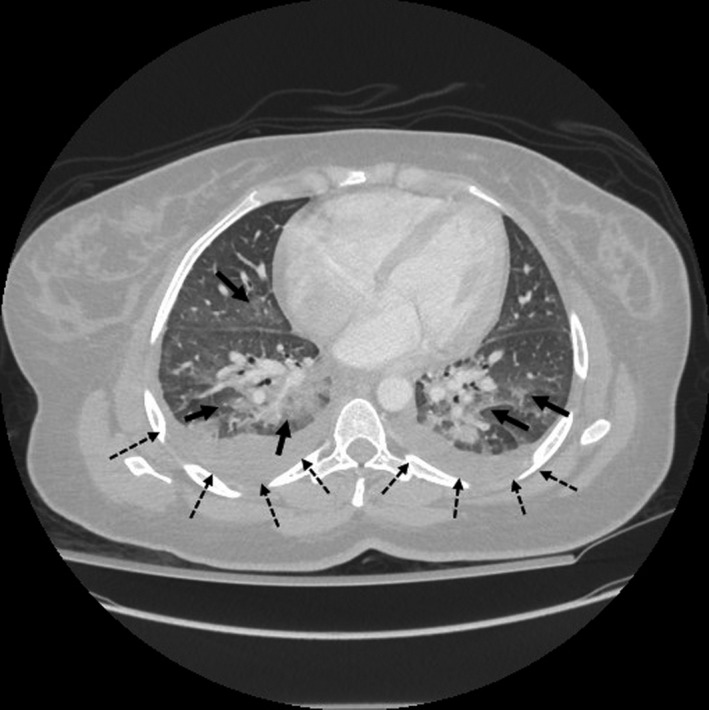
Computed tomography scan of the chest of a 37‐year‐old woman with Lemierre's syndrome shows multifocal pneumonia (solid arrows) with bilateral pleural effusion (dashed arrows).

On hospital day 2, the patient became progressively more tachycardic (heart rate, 135 b.p.m.) and tachypneic (22 breaths/min), developed worsening leukocytosis (19.5 K/μL), and became febrile (38.8°C) and hypoxic (SpO_2_ 88% on room air). At this time, i.v. cefepime and metronidazole were started empirically. She was transferred to the intensive care unit. A CT scan of the chest, abdomen, and pelvis with i.v. contrast showed development of bilateral pleural effusions, with scattered ground glass and consolidative airspace opacities of both lower lobes of the lungs. Of note, pancreatitis was resolved. Her serum lipase was normalized. Blood cultures from the day of admission were found to be positive for *F. necrophorum*. Further questioning into the patient's symptoms revealed that she had suffered from a sore throat 1 week prior to her other symptoms when she presented to the emergency department, and that she was still experiencing right‐sided lateral neck pain. A bedside ultrasound showed a partial thrombus in the right internal jugular vein, which was confirmed on formal Doppler studies (Fig. 2A). A CT scan of the soft tissue of the neck with contrast showed a thrombus within the right internal jugular vein (Fig. 2B,C) as well as new confluent opacities in the upper lungs bilaterally, consistent with multifocal pneumonia suspected to be due to septic emboli (Fig. 3). At this point, the diagnosis of LS was confirmed. Intravenous metronidazole was continued and anticoagulation therapy was initiated with heparin infusion followed by warfarin. The patient's symptoms improved. On discharge, metronidazole was switched to i.v. for 4 weeks on discharge followed by 2 weeks of oral amoxicillin‐clavulanate. She successfully completed her treatment without complications.

## Discussion

Named after Andre Lemierre's initial report in 1936,^1^ LS continues to be a clinical challenge for early diagnosis due to its various manifestations secondary to metastatic septic emboli. It is a very rare entity as there have been less than 160 published cases since his case series.^2^

Pathogenesis is associated with pharyngitis affecting the palatine tonsils or peritonsillar tissue.^7^ Other sources include upper and lower respiratory, mastoiditis, laryngitis, dental, paranasal sinusitis, parotitis, orbital, gastrointestinal, and lip piercing.^2^ These infections invade to the carotid sheath and cause IJV septic thrombophlebitis within 1–3 weeks.^7^, ^8^ The most common causative organism is *F. necrophorum* or other Fusobacterium species.^9^

The diagnosis of LS tends to be delayed until the blood culture reveals anaerobic Gram‐negative rods. The IJV thrombophlebitis presents as unilateral cervical tenderness that mimics lymphadenopathy. Metastatic septic emboli are common in lung (85%) and large joints (16–26%).^2^, ^3^ Other metastatic diseases include soft tissue abscess, cutaneous lesions, pyomyositis, splenic and liver abscess, osteomyelitis, endocarditis, pericarditis, renal abscess, hemolytic uremic syndrome, and central nervous system complications including encephalopathy, meningitis, cerebral infarctions, epidural abscess, and brain abscess.^3^, ^7^, ^8^ A CT scan of the neck with i.v. contrast is the imaging method of choice for the diagnosis of LS. It shows not only the IJV thrombosis but also other complications such as pulmonary emboli.^7^

Our case presented as acute pancreatitis and the diagnosis was delayed 2 days until the blood culture revealed *F. necrophorum*. In retrospect, the patient complained of oropharyngeal symptoms including neck pain and cough which were typical for LS, but not for acute pancreatitis. It was a pitfall for us to label the patient as having acute pancreatitis without investigating the cause of oropharyngeal symptoms that were not typical for acute pancreatitis. To date, there are no published data reporting the relationship between acute pancreatitis and LS. As other reports suggested that septic emboli could metastasize to any part of the body through the blood stream,^2^, ^3^, ^7^, ^8^ there was a possibility that the patient's acute pancreatitis was caused by the metastatic septic emboli as a part of LS. The learning points from this case are that LS and acute pancreatitis can occur simultaneously and, most importantly, assuming one diagnosis without paying attention to the symptoms not compatible with it (in our case, oropharyngeal symptoms not compatible with acute pancreatitis) can lead to the delayed diagnosis of LS.

## Conclusion

We reported a case of LS complicated with acute pancreatitis potentially caused by metastatic septic emboli. Oropharyngeal symptoms were the early indicator of LS despite prominent symptoms of acute pancreatitis. Therefore, it is important to have a high index of suspicion for early diagnosis and appropriate treatment.

## Disclosure

Source(s) of financial support: None.

Approval of the research protocol: Not applicable.

Informed consent (if applicable): Yes.

Registry and the registration no. of the study/trial: Not applicable.

Animal studies (if applicable): Not applicable.

Conflict of interest: None.
